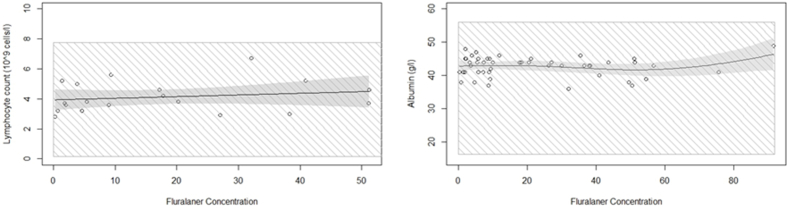# Pharmacokinetics and safety of topical fluralaner in koalas *(Phascolarctos cinereus)*

**DOI:** 10.1016/j.ijppaw.2024.100999

**Published:** 2024-09-24

**Authors:** Ellyssia T. Young, Jessica McKelson, Daniel Kalstrom, Lachlan Sipthorp, Leanne Wicker, Damien Higgins, Caroline Marschner, David S. Nichols, David Phalen, Aaron C. Greenville, Scott Carver

**Affiliations:** a School of Life and Environmental Sciences, The University of Sydney, Sydney, New South Wales, Australia; b Phillip Island Nature Park, Cowes, Victoria, Australia; c Zoos Victoria, Victoria, Australia; d Wildvet, Australia; e Sydney School of Veterinary Science, The University of Sydney, Sydney, New South Wales, Australia; f Central Science Laboratory, University of Tasmania, Hobart, Tasmania, Australia; g Odum School of Ecology, University of Georgia, Athens, GA, USA

**Keywords:** Fluralaner, Sarcoptic mange, *Sarcoptes scabiei*, Koala, Safety, Pharmacokinetics, *Phascolarctos cinereus*

## Abstract

Sarcoptic mange (etiological agent *Sarcoptes scabiei*) is among the most important parasitic diseases of some marsupial species and has been an emerging disease of koalas, causing welfare and conservation implications. Fluralaner (Bravecto® MSD Animal Health), an ectoparasiticide of the isoxazoline class, has been demonstrated as a long-lasting and efficacious chemotherapeutic agent against sarcoptic mange in multiple mammal species and may also be beneficial for impacted koalas. Here, we evaluated the pharmacokinetics and clinical safety of fluralaner in koalas. Healthy captive individuals were treated topically with 85 mg/kg fluralaner administered to the interscapular epidermis. Following treatment, fluralaner was detected in plasma using ultra-performance liquid chromatography and tandem mass-spectrometry over a 12-week period. The mean maximum plasma concentration (C_max_) was 66.4 ng/mL; mean time was C_max_ of 2.71 days; plasma elimination half-life (T_1/2_) was 30.91 days; and mean residence time (MRT) was 27.38 days. Haematological, blood biochemical, animal husbandry and clinical observations, over the same time period, demonstrated fluralaner was well tolerated. Overall, this research suggests fluralaner is a safe and long-lasting chemotherapeutic agent that may be efficacious against *S. scabiei* in koalas. Further research focussed on quantifying efficacy in captive and field settings, and across a range of disease severities would be valuable.

## Highlights

• Koalas were treated with fluralaner that remained plasma-detectable for 12 weeks.
• Standard safety measures demonstrate fluralaner was well tolerated.
• Fluralaner is safe, and long-lasting and may be efficacious against *Sarcoptes scabiei*.

## Graphical abstract

**Figure:**
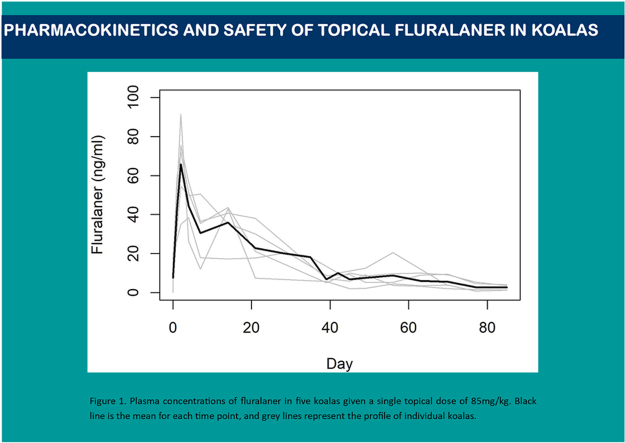

## 1 Introduction

Most therapeutics used on wildlife are developed in domestic animals. Accordingly, pharmacokinetic and safety (as measured by haematology, serum biochemistry and clinical observation) trials are important in determining the relative value of therapeutic agents being repurposed for use in wildlife disease management (Toutain et al., 2010). The value of pharmacokinetic and safety studies is particularly notable for marsupials, as there is comparatively little known about the pharmacokinetics or safety of many pharmaceutical agents used, and inferences are often drawn from studies on eutherian mammals (Kinzer, 1983; León-Vizcaíno et al., 2001; Martin et al., 2019). However, due to species-specific variation in physiological processes of absorption, distribution, metabolism and excretion, such extrapolation from domestic eutherian mammals to marsupials may have limitations (Mayer et al., 2006; Death et al., 2011). Therefore establishing specifes-specific safety and pharmacology of chemotherapeutic drugs is an integral part of developing safe and effective disease management.

Sarcoptic mange (etiological agent *Sarcoptes scabiei*) is one of the most important parasitic diseases affecting some Australian marsupials (Skerratt et al., 1999) and mammals more generally (Escobar et al., 2022). It is generally accepted that sarcoptic mange was introduced to Australia via humans and their domestic animals (dogs and possibly also agricultural and invasive animals) during European colonialism and has likely resulted in multiple spillover events, spreading over a wide geographic area (Fraser et al., 2017). In some native marsupials, sarcoptic mange is enzootic (e.g., bare-nosed wombat *Vombatus ursinus*), and in others it appears as sporadic cases or occasional outbreaks (e.g., southern hairy-nosed wombat *Lasiorhinus latifrons*, wallabies *Wallabia bicolor* and *Notamacropus agilis*, and possums *Pseudocheirus peregrinus*) (Domrow, 1992; McLelland and Youl, 2005; Wicks et al., 2007; Holz et al., 2011; Botten et al., 2022). In koalas (*Phascolarctos cinereus)* and the western brown bandicoot (*Isoodon fusciventer*) sarcoptic mange may be regionally becoming endemic (Speight et al., 2017; Botten et al., 2022). Clinical presentation varies among host species, but common signs include alopecia, hyperkeratosis, pruritis, and erythema (Bornstein et al., 2001; Pence and Ueckermann, 2002; Escobar et al., 2022). Species-specific behavioural ecology is a key contributor to disease occurrence and persistence (Browne et al., 2022). Furthermore, *S. scabiei* is treatable using a range of pharmacological agents, including ivermectin (Skerratt, 2003), moxidectin (Death et al., 2011) and fluralaner (Van Wick and Hashem, 2019; Wilkinson et al., 2021).

Koalas are facing numerous threats and are currently classified by the IUCN as Vulnerable (Woinarski and Burbidge, 2020). Among the emerging threats is sarcoptic mange (Tompkins et al., 2015). Koalas develop the more severe disease form termed crusted mange (Speight et al., 2017; Næsborg-Nielsen et al., 2022), characterised by severe parakeratotic lesions on the feet and lower regions of the limbs which can extend onto the face and ventral side of the animal, with significant crusting and fissuring of the epidermis resulting in open wounds, increased risk of secondary infections, loss of body condition, and death (Speight et al., 2017). Sarcoptic mange has been documented in koalas sporadically across their range in Victoria, South Australia and New South Wales (Obendorf, 1983; Canfield, 1987; Speight et al., 2017, 2018), but there is limited knowledge about epizootiology. Since mange causes severe disease that results in significant and prolonged welfare impact in koalas, there is an urgent need to find solutions to this issue (Carver et al., 2022).

Fluralaner (Bravecto® MSD Animal Health) is a new class of antiparasitic compound originally developed as a flea and tick treatment for dogs and cats (Kilp et al., 2016), and has been shown to be an efficacious and long-lasting treatment against sarcoptic mange in domestic animals and wildlife, such as wombats (Wilkinson et al., 2021), rabbits (Singh et al., 2022) and black bears (Van Wick and Hashem, 2019). Key properties of fluralaner as a treatment for sarcoptic mange are a rapid onset of effect (Kilp et al., 2016), extended half-life and duration of action (e.g., 1–3 months in bare-nosed wombats) relative to other therapeutic options (Wilkinson et al., 2021). Owing to the rapid onset and extended duration of action, fluralaner also has potential to interrupt the 11–14 day lifecycle of *S. scabiei* on the host (Bornstein et al., 2001) and prevent reinfestation of individuals from environmental sources and other animals (Rowe et al., 2019; Wilkinson et al., 2021; Browne et al., 2022). Thus, use of fluralaner has potential to minimise the number of drug administrations for achieving parasite control and clinical resolution. Importantly, fluralaner has also been reported to have a wide safety margin in domestic and free-ranging animals (Gassel et al., 2014; Walther et al., 2014; Prohaczik et al., 2017; Fisara et al., 2018; Van Wick and Hashem, 2019). However, the pharmacokinetic profile of fluralaner has been demonstrated to vary among species (Kilp et al., 2016; Wilkinson et al., 2021), suggesting species-specific metabolic and body fat effects are possible (Martin et al., 2018; Wilkinson et al., 2021).

In this study, we sought to establish the pharmacokinetic profile and safety of fluralaner in koalas. Because wildlife are more likely to be treated due to presence of mange signs, rather then as preventative medicine as in domestic animals, so a higher dose is often necessary, but within safety limits (Taenzler et al., 2016; Wilkinson et al., 2021). Thus we focussed on the pharmacokinetics and safety of fluralaner in koalas at a single topical dose of 85 mg/kg, comparable to what has been investigated in bare-nosed wombats (Wilkinson et al., 2021). Overall, we find fluralaner to be long-lasting and well tolerated in koalas.

## 2 Methods

### 2.1 Trial and sample collection

Five clinically healthy adult koalas (2 males and 3 females, ages ranging from 4 to 7 years) were enlisted in this study. All were residents at the Phillip Island Nature Park and were housed in free-range enclosures. This study was approved by University of Sydney Animal Ethics Committee and Phillip Island Nature Park Animal Ethics (AEC Project number 7.2021). Average body weight of female koalas was 8.5 kg and 10.4 kg for males. Based on a recent fluralaner pharmacokinetic study in bare-nosed wombats (Wilkinson et al., 2021, 2024) an 85 mg/kg dosage was adopted, and spot-on application was chosen as the most feasible commercially available (Bravecto®) method of administering fluralaner to captive held koalas. All procedures were conducted by a wildlife veterinarian (L. Wicker) between March and June 2022. Daily monitoring by park staff included faecal output, appetite and demeanour monitoring, weekly health assessments by park staff and the supervising veterinarian, including pre-examination assessment of movement and demeanour, measurement of body weight, physical examination under manual restraint, and collection of blood for assessment of haematological and blood biochemical parameters.

On day 0 of this trial, each animal was anaesthetised for a thorough assessment of animal health (including body weight, body condition score, and full physical examination) collection of blood via cephalic venipuncture, and topical application of 85 mg/kg fluralaner (Bravecto® Spot-on) to the interscapular epidermis. Anaesthesia of the first koala was induced via intramuscular injection of alfaxalone 2 mg/kg and medetomidine 40 μg kg^−1^, reversed with atipamezole at 0.16 mg kg-1 at the end of the procedure, as described Downey et al. (2020). For the remaining four koalas, anaesthesia was induced via mask delivery of 5% isoflurane in oxygen and maintained via mask delivery of 1–2% isoflurane in oxygen, a regimen which resulted in more stable anaesthesia and more rapid recovery following the cessation of the procedure. Additional blood samples were collected from each koala while they were manually restrained on day 0 at times 0, 1, and 4 h after treatment and on days 1, 2, 4, 7, 14, 21, 28, 35, 42, 49, 56, 63, 70, 77 and 85.

Each blood sample was aliquoted into 1 ml subsamples in separate ethylenediaminetetraacetic (EDTA) blood collection tubes for pharmacokinetic and haematological analyses, a lithium heparin tube for biochemical analysis, and a fresh blood smear made within 1 h of blood collection to support haematological evaluation. Aliquots intended for biochemical analysis were stored at 4 °C until analysis within 24 h of blood collection. Aliquots intended for pharmacokinetic analysis were centrifuged to extract the plasma from the whole blood sample, and plasma was stored at −20 °C until submission to the laboratory.

Ultra-high performance liquid chromatography-tandem mass spectrometry (UPLC-MS/MS) using a Waters Acquity H-class UPLC system (Waters Corporation, Milford, MA) was undertaken to determine the fluralaner concentration in plasma. Plasma samples underwent liquid-liquid extraction and protein precipitation prior to the analysis of solvent extracts as described by Wilkinson et al. (2021) with the following modification. For quantitative determination, stable isotope dilution was employed utilising the addition of ^13^C_4_,^2^H_3_-Fluralaner (Alsachim, France) to each plasma sample prior to extraction (41.6 μg per sample). Chromatography was performed using an Acquity BEH C18 VanGuard pre-column (5.0 × 2.1 mm, 1.7 μm) and an Aquity BEH C18 column (2.1 × 100 mm × 1.7 μm) (Waters Corporation). The UPLC was operated with a mobile phase consisting of 0.1% (v/v) Formic acid (Solvent A) and Acetonitrile (Solvent B). Elution was using a gradient. Initial conditions were 20% B before a gradient to 95% B over 4 min, which was held for 2 min. The system was returned to initial conditions at 6.5 min and re-equilibrated for 3 min. The flow rate was 0.35 ml/min and the column was held at 45 °C. Injection volume was 2 μl. The UPLC was coupled to a Waters Xevo TQ triple quadrupole mass spectrometer (Waters Corporation). Analyses were undertaken using multiple reaction monitoring (MRM) in negative electrospray ionisation mode, with 2 MRM Transitions monitored for Fluralaner as described by Wilkinson et al. (2021). Electrospray ionisation was performed with a capillary voltage of 2.5 kV, and individual cone voltages and collision energies for each MRM transition, as described below. The desolvation temperature was 450 °C, nebulising gas was nitrogen at 950 l/h and cone gas was nitrogen at 100 l/h. MRM transition dwell times were 120 msec. Quantitation was undertaken using matrix matched (koala plasma) external calibration standards as described by Wilkinson et al. (2021).

Plasma biochemistry and electrolyte analysis was conducted in house using a VetScan VS2 Chemistry analyser, Comprehensive Diagnostic Profile (Zoetis Inc. Australia) and full haematological analysis (automated cell count and blood smear evaluation) were conducted at Gribbles Veterinary Pathology, Australia. Biochemical and haematological results were clinically interpreted against expected values for the species and analysed against publish species reference ranges to provide a clinical assessment of animal health, identify changes in values for each parameter over time, and interrogated to assess for signs of drug toxicity.

### 2.2 Data analyses

All statistical analyses were undertaken using R (R version 4.2.2).

Non-compartmental methods (R packages ‘pk’ and ‘linpk’ (Jaki and Wolfsegger, 2011; Rich, 2022)) were also used to calculate fluralaner pharmacokinetic values, maximum recorded plasma concentrations (C_max_), time to C_max_ (T_max_), area under curve (AUC), plasma elimination half-life (t_1/2_) and mean residence time (MRT).

The dynamics of individual haematology variables, biochemistry values, and body weight in relation to time and fluralaner concentration were assessed using generalised additive mixed models (GAMMs) using the R package ‘mgcv’ (Wood, 2011), with koala ID as the random effect. GAMMs were used because it enabled non-linear changes in the response variables over time to be modelled as a spline function. Finally, the relative ‘health relevance’ of haematology and biochemistry values were also conservatively assessed in relation to available reference range information, acknowledging current published reference ranges are based on small numbers of geographically restricted koalas (Canfield et al., 1989; Pye et al., 2012; Speight et al., 2014; Fabijan et al., 2020), and assessed against values for each parameter listed in the Zoo Information Management System (ZIMS) (Species360 Zoological Information Management System (ZIMS), 2022).

## 3 Results

The five koalas were assessed as being clinically healthy based on thorough physical examination and the results of blood biochemistry and haematology obtained on Day 0. Topical fluralaner was administered to the interscapular region at a dosage of 85 mg/kg and monitored continuously for 12 weeks. There were no deleterious changes in gross behaviour, appetence, faecal production or body mass observed. All koalas remained alert, normally responsive, maintained appetence and with normal faecal output for the duration of the study. Body mass exhibited a slight increase throughout the study period in relation to time, but not fluralaner concentration (time – F = 3.212, P = 0.08; fluralaner plasma concentration – t = 0.483, P = 0.631) (see Appendix 2 Figure 2).

### 3.1 Pharmacokinetics

After topical administration, fluralaner was detected in the plasma, initially exhibiting a rapid rise in concentration and then slow decay over the 12-week period (Fig. 1, F = 16.36, P < 0.001). Pharmacokinetic calculations showed fluralaner had an average: C_max_ 66.4 ng/mL, T_max_ 2.71 days, AUC 1270.87 day∗ng/ml, T_1/2_ 30.91 days, and MRT 27.38 days.

**Fig. 1:**
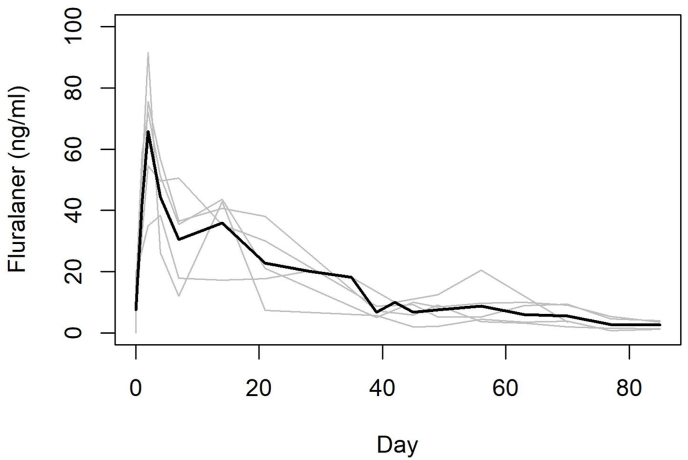
Plasma concentrations of fluralaner in five koalas given a single topical dose of 85 mg/kg. Black line is the mean for each time point, and grey lines represent the profile of individual koalas.

### 3.2 Safety

**figs3:**
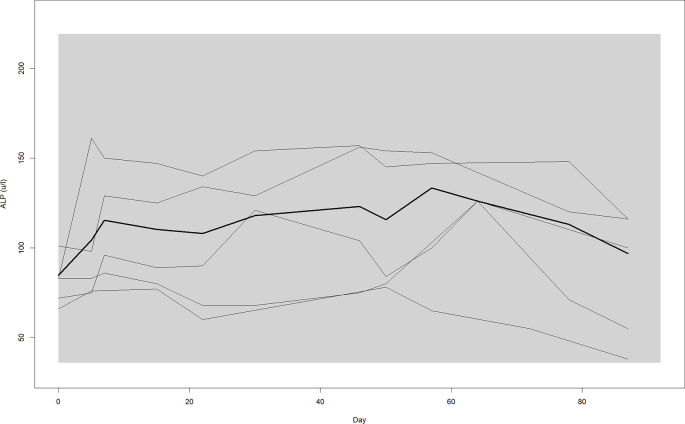


**figs4:**
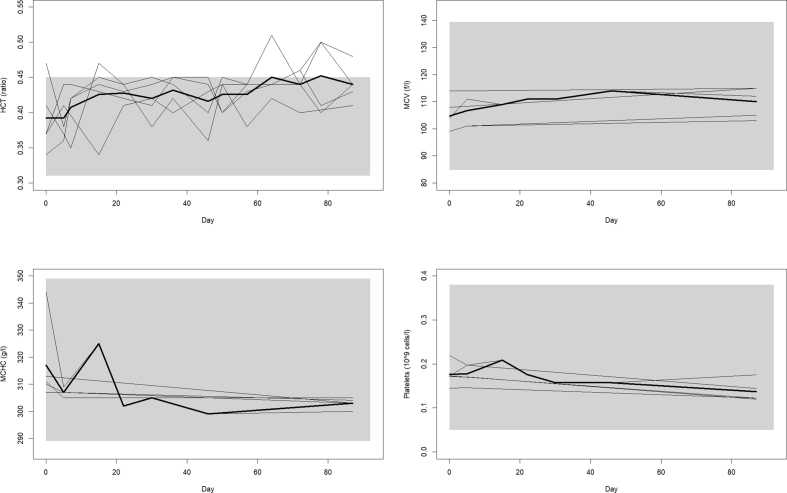


**figs5:**
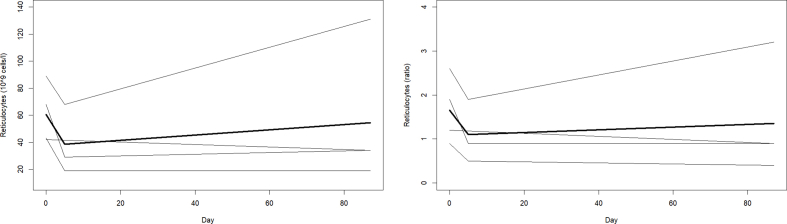


**figs6:**
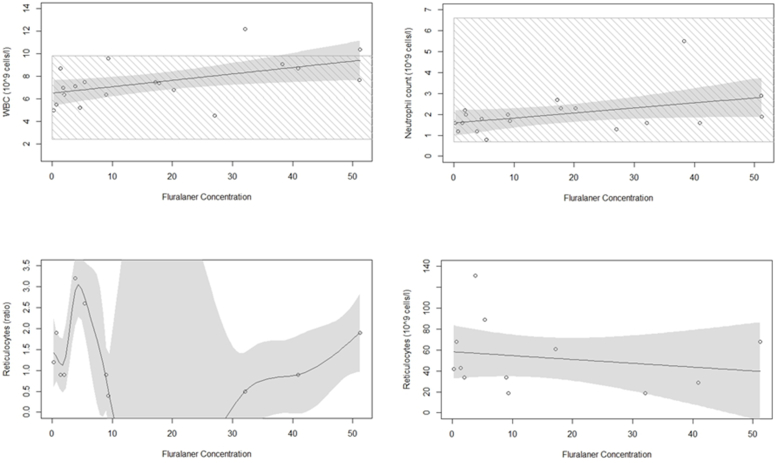


Overall, no deleterious drug induced effects of fluralaner on haematology and biochemistry values were evident over the 12-week trial (Table 1, Table 2). Increases over time, predominantly within reference range information (Table 3, Table 4, Appendix 2 Figs. 3–6), were documented for total protein, globulin, haematocrit, and mean corpuscular volume, with only a slight increase observed in albumin. Significant decreases over time, again predominantly within reference range information were observed for glucose, alkaline phosphatase, platelets and mean corpuscular haemoglobin concentration.

**Table 1:** Plasma biochemistry reference intervals for healthy koalas (*Phascolarctos cinereus*) used in this study relative to the mean, minimum and maximum values obtained from koalas over the duration of this trial (n = 5).

Biochemical parameter	Reference interval^a^	Trial (n = 5)	
		Average	Min-Max
Sodium (mmol/l)	132–147	143.8	134–157
Calcium (mmol/l)	1.47-5.47	3	1.0–3.9
Phosphorus (mmol/l)	0.12-2.02	1.3	0.71-1.81
Glucose (mmol/l)	0.1–8.35	4.9	3.0–6.6
Blood urea nitrogen (mmol/l)	0.8–32.6	3.4	0.9-6.0
Creatinine (μmol/l)	4.0–3249	74.6	38–113
Alanine aminotransferase (u/l)	0–983	12.5	5.0–24
Alkaline phosphatase (u/l)	36–219	102.9	38–161
Total Bilirubin (μmol/l)	0–13.5	4.2	3.0–9.0
Total protein (g/l)	15.9–85.4	71.6	58–84
Albumin (g/l)	16.3–56	42.8	36–49
Globulin (g/l)	15–45	28.3	20.0–41.0

a (Canfield et al., 1989; Pye et al., 2012; Speight et al., 2014; Species360 Zoological Information Management System (ZIMS), 2022).

**Table 2:** Haematological reference intervals from health koalas (*Phascolarctos cinereus*), relative to the mean, minimum and maximum values obtained from koalas over the duration of this trial (n = 5) located at Phillip Island Nature Park, Victoria, Australia.

Haematological parameter	Reference interval^a^	Trial (n = 5)	
		Average	Min-Max
RBC (10^12 cells/l)	2.29-4.43	3.78	3.16-4.09
HGB (g/l)	77–140	125.53	106–133
HCT (ratio)	0.31-0.45	0.42	0.34-0.51
MCV (fl)	84.7–139.5	108.11	99–115
MCH (pg)	28.9–41.6	33.34	30.8–35.7
MCHC (g/l)	289–373	308.83	299–344
Reticulocytes (10^9 cells/l)	Insufficient data	53	19–131
Reticulocytes (ratio)	Insufficient data	1.39	0.4-3.2
Nucleated red blood cells (/100WBCs)	0.0–36	7.91	1.0–12
Platelets (10^9 cells/l)	0.05-0.38	21.92	0.12–219
WBC (10^9 cells/l)	2.4–9.84	7.51	4.5–12.2
Lymphocyte count (10^9 cells/l)	0.17-7.77	4.13	2.8-6.7
Monocyte count (10^9 cells/l)	0.00–1.08	0.34	0.1-0.8
Neutrophil count (10^9 cells/l)	0.7-6.62	2.01	0.8-5.5
Band count (10^9 cells/l)	0–0.281	0	0–0
Eosinophil count (10^9 cells/l)	0.0–1.1	0.9	0–3.4
Basophil count (10^9 cells/l)	0.00–0.11	0.02	0–0.1
Lymphocyte (%)	10.0–92.2	55.68	33–70
Monocyte (%)	0.0–10.0	4.68	1.0–8.0
Neutrophil (%)	4.4–86.1	27.58	11.0–60
Band (%)	0.0–5.0	0	0–0
Eosinophil (%)	0–4.00	10.89	0–33
Basophil (%)	0.0–3.00	0.16	0.0–1.0

a (Canfield et al., 1989; Fabijan et al., 2020; Species360 Zoological Information Management System (ZIMS), 2022).

**Table 3:** Generalised additive mixed model outputs for biochemical parameters for all koalas (*Phascolarctos cinereus*) (n = 5) involved in the study and located at Phillip Island Nature Park, Victoria, Australia.

Biochemical parameter	Time		Fluralaner concentration	
	F	P	t	P
Sodium (mmol/l)	1.575	0.284	0.171	0.865
Creatinine (μmol/l)	3.648	0.063	0.491	0.626
Calcium (mmol/l)	2.013	0.164	1.528	0.134
Blood urea nitrogen (mmol/l)	0.183	0.671	0.877	0.386
Glucose (mmol/l)	7.834	0.008	0.685	0.497
Total protein (g/l)	17.69	<0.001	2.139	0.385
Alanine aminotransferase (u/l)	0.392	0.534	0.864	0.393
Alkaline phosphatase (u/l)	5.918	0.003	1.923	0.062
Total Bilirubin (μmol/l)	0.003	0.96	0.171	0.865
Albumin (g/l)	10.32	0.003	2.142	0.038
Globulin (g/l)	10.55	0.002	1.805	0.078

**Table 4:** Generalised additive mixed model outputs for haematological parameters for all koalas (*Phascolarctos cinereus*) (n = 5) involved in the study and located at Phillip Island Nature Park, Victoria, Australia.

Haematological parameter	Time		Fluralaner concentration	
	F	P	t	P
Haemoglobin (g/l)	4.29	0.06	2.08	0.06
Haematocrit (ratio)	8.51	0.01	0.01	0.99
Red blood cells (10^12 cells/l)	2.41	0.14	1.72	0.11
Mean corpuscular volume (fl)	6.68	0.01	1.46	0.17
Mean corpuscular haemoglobin (pg)	0.20	0.66	0.34	0.74
Mean corpuscular haemoglobin concentration (g/l)	4.59	0.05	0.78	0.45
Reticulocytes (10^9 cells/l)	6.49	0.03	2.84	0.02
Reticulocytes (ratio)	14.23	0.01	3.68	0.01
Nucleated red blood (/WBCs)	4.78	0.07	1.04	0.34
Platelets (10^9 cells/l)	5.44	0.01	0.56	0.59
White blood cells (10^9 cells/l)	2.23	0.16	4.47	<0.01
Lymphocyte count (10^9 cells/l)	0.55	0.39	1.98	0.07
Monocyte count (10^9 cells/l)	0.91	0.36	0.70	0.50
Neutrophil count (10^9 cells/l)	1.55	0.15	2.12	0.05
Eosinophil count (10^9 cells/l)	1.84	0.20	1.30	0.21
Lymphocytes (%)	0.14	0.71	2.20	0.04
Monocytes (%)	0.64	0.44	0.50	0.62
Neutrophils (%)	0.96	0.34	0.89	0.39
Eosinophils (%)	2.92	0.11	0.78	0.45

While some changes in these variables over time were observed, we also looked at whether these variables changed in relation to fluralaner concentration (Table 3, Table 4, Appendix 2 Figs. 7–8). Changes in haematology and blood biochemistry was observed in relation to fluralaner plasma concentration (Table 3, Table 4, Appendix 2 Figs. 7–8). A decrease in reticulocytes was observed whereas albumin, lymphocyte count, neutrophil count, and white blood cells increased, and reticulocytes ratio showed high variability with an overall increase with increased plasma fluralaner concentration.

The remaining parameters did not change significantly over time or fluralaner concentration and were mostly within reference intervals (Table 1, Table 2). Some koalas showed occasional variation in biochemical parameters outside of reference ranges (see Appendix 2 Figs. 3–8) but were generally concluded to be not clinically indicative of drug toxicity or were within the expected error range of measurements. Some parameters were omitted from analysis due to a high number of suspected measurement errors (potassium) and band count was omitted from modelling as none were detected in any of the koalas.

## 4 Discussion

Wildlife medicines are largely derived from those developed for domestic animals, with pharmacokinetic properties and safety assumed by extrapolation (Kinzer, 1983; León-Vizcaíno et al., 2001; Govendir, 2018; Martin et al., 2019). However, differences in drug dynamics and safety occur among species, as well as conditions of use (e.g., prophylactic vs. therapeutic application) (Toutain et al., 2010; Takano et al., 2023). Here we undertook an analysis of the pharmacokinetics and safety of the ectoparasiticide fluralaner in five healthy captive adult koalas. Our key findings are: *i)*. fluralaner was detectable in plasma at concentrations that have been shown to be therapeutic against *S. scabiei* mites in related species the wombat, *ii)*. fluralaner persisted in plasma at quantifiable levels for at least 12 weeks post treatment, and *iii)* a single topical 85 mg/kg dose of fluralaner was safely tolerated in the koalas treated (as assessed by complete cell counts, repeated blood biochemistries, and clinical observation). These results suggest fluralaner may be a viable long-lasting treatment for sarcoptic mange in koalas.

The pharmacokinetic profile of fluralaner in koalas demonstrated a relatively long duration of action for an ectoparasiticide. In comparison, other therapeutic agents that have been used to treat koalas for sarcoptic mange typically exhibit shorter durations of protection and often require repeated treatments, including malathion (Barker, 1974), amitraz (Brown et al., 1982), ivermectin (Speight et al., 2017), and moxidectin (Young et al., 2024). Our findings of a relatively long T_1/2_ and MRT for fluralaner are broadly consistent for trials of isooxazolines in other mammal species (Kilp et al., 2016; Wilkinson et al., 2021), and may confer distinct therapeutic advantages for treated koalas, relative to the aforementioned therapeutic agents. We recognised three key areas where fluralaner may provide significant value for koalas: 1) fluralaner is known to induce rapid killing of mites on the host (Beugnet et al., 2015; Wilkinson et al., 2021); 2) diminished requirements to re-treat mange affected koalas in care, resulting in less need for handling and restraint associated with treatment, and thus less stress to the individual; and 3) fluralaner has a high potential for breaking the transmission cycle of the mite both on the host and in the environment owing to this long duration of protection.

The pharmacokinetics of fluralaner in koalas was similar to other mammal species, in that it is longer lasting than most macrocyclic lactones (Wilkinson et al., 2021). Our data along with that of others demonstrates that pharmacokinetic parameters should be expected to vary significantly between species (Kilp et al., 2016; Wilkinson et al., 2021). In the current study, the mean C_max_ was nearly 6 times higher in koalas (66.4 ng/ml) as compared to wombats given the same dose rate (16.4 ng/ml), but was still substantially lower than the C_max_ determined in dogs (1698 ng/ml) and cats (2399 ng/ml) (Kilp et al., 2016; Wilkinson et al., 2021). Additionally, the T_1/2_ was shorter in koalas (31 days) than in wombats (166.5 days, but note n = 2), but longer than observed in cats (12 days at 80 mg/kg dose) and dogs (17 days at 50 mg/kg dose) (Kilp et al., 2016; Wilkinson et al., 2021). Fluralaner demonstrated long systemic persistence in koalas, and the previous studies mentioned above observed parasite extermination and efficacy for 3 months or longer, with shorter fluralaner half-life. So, it is likely that this extended duration of action would also be observed in koalas.

Overall, the pharmacokinetic profiles of topical fluralaner in koalas was similar to that reported in wombats (Wilkinson et al., 2021), a closely related species. This is somewhat expected as wombat and koala diets, habitats and adaptations to survive in Australia has shaped their physiological processes (Govendir, 2018). The underlying explanations for the smaller differences in pharmacokinetics are not yet clear, but they may relate to species specific differences in absorption, distribution, metabolism and excretion processes (Toutain et al., 2010). Our pharmacokinetic calculations suggest that measurable amounts of fluralaner persists in plasma for at least 85 days. The duration of protection against S. scabiei remains to be understood, and further research clarifying drug efficacy is warranted. Further studies into the absorption and distribution of fluralaner into other tissues, particularly adipose tissue since fluralaner is lipophilic, is required to gain a more complete understanding of fluralaner pharmacokinetics in koalas.

Koalas have a specialist folivorous diet primarily composed of eucalyptus foliage (Moore and Foley, 2005). Eucalyptus leaves have comparatively low nutrient content (Cork and Sanson, 1991) and contain plant secondary metabolites (PSMs), including some which are cytotoxic (Moore and Foley, 2005). Koalas behaviour and physiology has co-evolved to cope with this specialist diet and use considerable amounts of their energy managing the absorption and excretion of these compounds (Freeland and Janzen, 1974; Krockenberger et al., 1998). Koalas have processes that limit the absorption and accelerate elimination of PSMs, xenobiotic compounds and toxins (Govendir, 2018), such as rapid hepatic metabolism (Govendir, 2018), and energy conservation strategies including low metabolism (Degabriele and Dawson, 1979; Nagy and Martin, 1985). These processes can reduce the efficacy of some therapeutic medicines (Govendir, 2018), and since fluralaner undergoes some metabolism in the liver (Kilp et al., 2016) these adaptations may influence how koalas absorb, distribute, metabolise and excrete potential toxins as well as PSMs, and can help to understand the pharmacokinetic profile of fluralaner observed in this study.

Previous studies on a taxonomically wide variety of species have shown that fluralaner has a wide safety margin in vertebrate species both wild and domestic (Walther et al., 2014; Prohaczik et al., 2017; Fisara et al., 2018; Van Wick and Hashem, 2019). In this study, koalas also tolerated fluralaner application at higher dosage than that recommended for domestic animals (Taenzler et al., 2016). Some changes in biochemical and haematological parameters were observed in the treated koalas, but never where the changes considered to be outside of the normal ranges for this species, nor were behavioural changes observed in these koalas, so we found no indication that fluralaner administered at the dosage used in this study had a negative impact on the treated animals. It is also important to note that haematology and blood biochemistry reference range information is limited in koalas (Canfield et al., 1989; Pye et al., 2012; Speight et al., 2014; Fabijan et al., 2020), and many wildlife species. Thus, the time zero data from our study provides valuable additional reference range information for koalas, and we recommend further reference range data collection would be useful for this threatened wildlife species.

Fluralaner is lipophilic in nature and therefore will distribute into fatty tissue (Committee for Medicinal Products for Veterinary Use, 2016). Koalas have low amounts of body fat (0.87–3.72%), relative to domestic species, such as dogs and cats (15–25% and up to 65%, respectively) (Laflamme, 1997; Toll et al., 2010; Witzel et al., 2014). The distribution of fluralaner into the adipose tissue can extend duration of action, as has been seen in dogs (Queiroga et al., 2021). While there were no clinical, physical or behavioural signs of toxicity demonstrated by the koalas in this study, monitoring is still needed to exhibit care regarding overdosing risks. Further, the sequestration of fluralaner into adipose tissue may explain differences in circulating fluralaner and persistence in the body.

Although our study cohort was limited to adult animals that were not reproducing, fluralaner had no impact on reproductive behaviour or success in dogs (Committee for Medicinal Products for Veterinary Use, 2016), or juvenile (Walther et al., 2014), or maternally dependent bare-nosed wombats (Wilkinson et al., 2021) and therefore is likely safe in reproductively active koalas and their young.

## 5 Conclusion

Here we demonstrate that healthy koalas safely tolerated transdermal application of fluralaner at a dose of 85 mg/kg and suggest that this drug may be efficacious in the treatment of sarcoptic mange in affected koalas for multiple weeks.

## Funding

This research was supported by an 10.13039/501100000923Australian Research Council Linkage Project(LP180101251) to SC and DP, an Australian Postgraduate Award to EW.

## Availability of data and materials

The datasets used and analysed during this study are available from the corresponding author on reasonable request.

## Ethics approval

Ethics approval was obtained from the University of Sydney animal ethics committee and Phillip Island Nature Park animal ethics committee (AEC Project number 7.2021).

## Consent for publication

Not applicable.

## CRediT authorship contribution statement

**Ellyssia T. Young:** Writing – original draft, Formal analysis, Data curation. **Jessica McKelson:** Writing – review & editing, Resources, Project administration, Methodology. **Daniel Kalstrom:** Investigation. **Lachlan Sipthorp:** Investigation. **Leanne Wicker:** Writing – review & editing, Validation, Methodology, Investigation, Data curation. **Damien Higgins:** Funding acquisition, Conceptualization. **Caroline Marschner:** Project administration, Funding acquisition. **David S. Nichols:** Methodology. **David Phalen:** Writing – review & editing, Supervision, Resources, Funding acquisition, Conceptualization. **Aaron C. Greenville:** Writing – review & editing, Supervision. **Scott Carver:** Writing – review & editing, Visualization, Supervision, Funding acquisition, Formal analysis, Conceptualization.

## Declaration of competing interest

The authors declare that they have no known competing financial interests or personal relationships that could have appeared to influence the work reported in this paper.

## Figures and Tables

**figs1:**
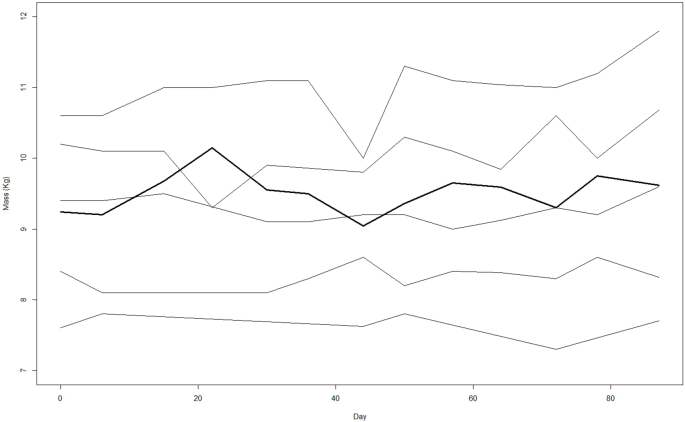


**figs2:**
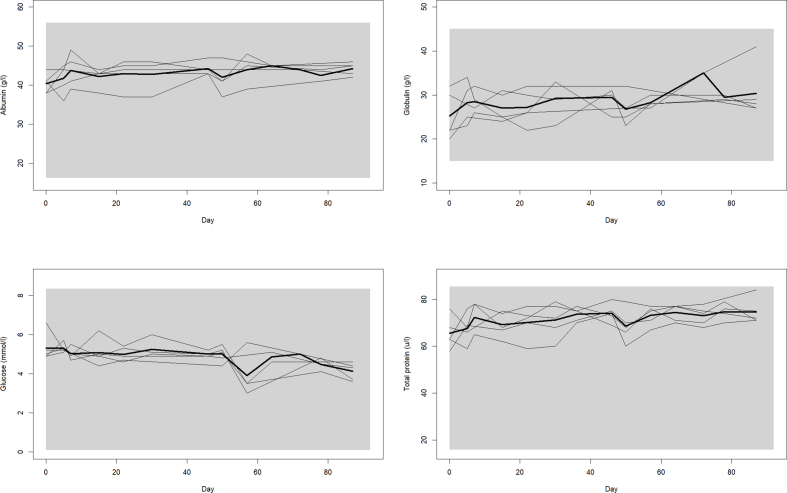


**figs7:**