# Supplementary material for: Pharmacokinetics and safety of topical fluralaner in koalas (Phascolarctos cinereus)
Source: Int J Parasitol Parasites Wildl. 2024 Sep 24;25:100999. doi: 10.1016/j.ijppaw.2024.100999 (PMC11993836; doi:10.1016/j.ijppaw.2024.100999)
Supplement: Multimedia component 1 [file mmc1.docx]

**SUPPLEMENTARY MATERIAL 1 - ULTRA-HIGH PERFORMANCE LIQUID CHROMATOGRAPHY-TANDEM MASS SPECTROMETRY (UPLC-MS/MS)**

*Sample preparation:*

Plasma samples (200 μl) were extracted with 780 μl of Methanol:Acetone (1:1, v/v) to which 20 μl of Surrogate Standard (^13^C_4_,^2^H_3_-Fluralaner; AlsaChim, France) solution in Methanol was added at a concentration of 2.08 ug/ml (Table 5). The extraction mix was vortexed for 30 sec and placed in a freezer (-20^o^C) for 60 min to allow protein precipitation. Extracts were then centrifuged at 15,000 rpm for 12 min at 4^o^C, and the supernatant transferred to a LC vial for analysis.

*Analysis:*

The UPLC instrument was a Waters Acquity H-class UPLC system (Waters Corporation, Milford, MA). Chromatography was performed using an Acquity BEH C18 VanGuard pre-column (5.0 x 2.1 mm, 1.7 μm) and an Aquity BEH C18 column (2.1 × 100 mm × 1.7 μm) (Waters Corporation). The UPLC was operated with a mobile phase consisting of 0.1% (v/v) Formic acid (Solvent A) and Acetonitrile (Solvent B).  Elution was using a gradient.  Initial conditions were 20% B before a gradient to 95% B over 4 min, which was held for 2 min. The system was returned to initial conditions at 6.5 min and re-equilibrated for 3 min. The flow rate was 0.35 ml/min and the column was held at 45^o^C. Injection volume was 2 μl.

The UPLC was coupled to a Waters Xevo TQ triple quadrupole mass spectrometer (Waters Corporation). Analyses were undertaken using multiple reaction monitoring (MRM) in negative electrospray ionisation mode, with 2 MRM Transitions monitored for Fluralaner and the Surrogate Standard, respectively. Electrospray ionisation was performed with a capillary voltage of 2.5 kV, and individual cone voltages and collision energies for each MRM transition, as described below. The desolvation temperature was 450°C, nebulising gas was nitrogen at 950 l/h and cone gas was nitrogen at 100 l/h. MRM transition dwell times were 120 msec.

**Table 5.** UPLC-MS/MS multiple reaction monitoring conditions employed for the analysis of fluralaner, and stable isotope labelled surrogate standard.

|  | Precursor | Product | Cone | Collision |
| --- | --- | --- | --- | --- |
| Analyte | [M-H]^-^ | [M-H]^-^ | Voltage (V) | Energy (V) |
|  | (m/z) | (m/z) |  |  |
|  |  |  |  |  |
|  |  |  |  |  |
| Fluralaner 1 | 554.0 | 534.0 | 44 | 17 |
| Fluralaner 2 | 554.0 | 494.0 | 44 | 23 |
|  |  |  |  |  |
|  |  |  |  |  |
| ^13^C_4_,^2^H_3_ – Fluralaner 1 | 561.0 | 501.0 | 44 | 17 |
| ^13^C_4_,^2^H_3_ – Fluralaner 2 | 561.0 | 541.0 | 44 | 23 |
|  |  |  |  |  |

**SUPPLEMENTARY MATERIAL 2 – CLINICAL PATHOLOGY TIME AND FLURALANER CONCENTRATION PLOTS**

See Figures 2-8.

**Figure 2.** Body mass of koalas (Phascolarctos cinereus) (n=5) over time (day) involved in this study and located at Phillips Island Nature Park, Victoria, Australia. Thick black line represents the mean, and the thin black lines represent individual koalas.

**Figure 3.** Observed values of albumin, globulin, glucose and total protein over time (day). The thick black line is the mean, the thin black lines represent individual koalas (Phascolarctos cinereus) (n=5) involved in this study and located at Phillip Island Nature Park, Victoria, Australia, and grey polygon indicates published reference ranges for koalas. (Canfield et al., 1989; Pye et al., 2012; Speight et al., 2014; Species360 Zoological Information Management System (ZIMS), 2022).

**Figure 4.** Observed values of alkaline phosphatase (ALP) over time (day). The thick black line is the mean, the thin black lines represent individual koalas (Phascolarctos cinereus) (n=5) involved in this study and located at Phillip Island Nature Park, Victoria, Australia, and grey polygon indicates published reference ranges for koalas. (Canfield et al., 1989; Pye et al., 2012; Speight et al., 2014; Species360 Zoological Information Management System (ZIMS), 2022).

**Figure 5.** Observed values of haematocrit (HCT), mean corpuscular volume (MCV), mean corpuscular haemoglobin concentration (MCHC) and platelets over time (day). The thick black line is the mean, the thin black lines represent individual koalas (Phascolarctos cinereus) (n=5) involved in this study and located at Phillip Island Nature Park, Victoria, Australia, and grey polygon indicates published reference ranges for koalas. (Canfield et al., 1989; Pye et al., 2012; Speight et al., 2014; Species360 Zoological Information Management System (ZIMS), 2022).

**Figure 6.** Observed values of reticulocytes and reticulocytes ratio over time (day). The thick black line is the mean, the thin black lines represent individual koalas (Phascolarctos cinereus) (n=5) involved in this study and located at Phillip Island Nature Park, Victoria, Australia. Reference range information was not available for these parameters. (Canfield et al., 1989; Pye et al., 2012; Speight et al., 2014; Species360 Zoological Information Management System (ZIMS), 2022).

**Figure 7.** Observed values of white blood cell count (WBC), neutrophil count, reticulocyte ratio, reticulocyte count against fluralaner concentration for all koalas (Phascolarctos cinereus) (n=5) involved in this study and located at Phillip Island Nature Park, Victoria, Australia. Black line is the predicted trend line, grey polygon represents the standard deviation, and grey hashed polygon represents published reference ranges. Reference range information was not available for reticulocytes and reticulocytes ratio parameters. (Canfield et al., 1989; Pye et al., 2012; Speight et al., 2014; Species360 Zoological Information Management System (ZIMS), 2022).

**Figure 8.** Observed values of lymphocyte count and albumin against fluralaner concentration for all koalas (Phascolarctos cinereus) (n=5) involved in this study and located at Phillip Island Nature Park, Victoria, Australia. Black line is the predicted trend line, grey polygon represents the standard deviation, and grey hashed polygon represents published reference ranges. (Pye et al., 2012; Fabijan et al., 2020; Species360 Zoological Information Management System (ZIMS), 2022).
